# Noninvasive Diagnosis of Chronic Exertional Compartment Syndrome With Shear Wave Elastography and Subharmonic‐Aided Pressure Estimation

**DOI:** 10.1002/jum.70030

**Published:** 2025-08-12

**Authors:** Corinne E. Wessner, Corbin Pomeranz, Rachel Blackman, Michael K. Hoy, Kristen Bradigan, Stephen A. Stache, Marc Harwood, Levon Nazarian, Flemming Forsberg

**Affiliations:** ^1^ Department of Radiology Thomas Jefferson University Philadelphia Pennsylvania USA; ^2^ Sidney Kimmel Medical College Thomas Jefferson University Philadelphia Pennsylvania USA; ^3^ Non‐Operative Sports Medicine Department Rothman Orthopaedic Institute Philadelphia Pennsylvania USA

**Keywords:** chronic exertional compartment syndrome, compartment pressure testing, shear‐wave elastography, subharmonic‐aided pressure estimation

## Abstract

**Objective:**

Chronic exertional compartment syndrome (CECS) is an underdiagnosed condition that affects young athletes. CECS is caused by increased compartmental pressure in the fascial spaces during exercise. CECS is diagnosed by direct pressure readings (in mmHg), which is a painful and invasive test. Noninvasive shear‐wave elastography (SWE) and subharmonic‐aided pressure estimation (SHAPE) using contrast‐enhanced ultrasound were compared to compartment pressure testing for the diagnosis of CECS.

**Methods:**

Ten healthy volunteers and five CECS patients enrolled in this prospective, IRB‐approved pilot study. Subjects were scanned pre‐ and post‐exercise using a modified LOGIQ E10 scanner (GE HealthCare). For SWE, muscle stiffness was expressed as median shear velocity over 12 measurements. Following intravenous infusion of Definity (3 mL in 50 mL of saline; Lantheus Medical Imaging) SHAPE power was optimized, and three 5‐second clips were acquired. SWE and SHAPE pre‐to‐post differences were calculated offline and compared to CECS pressure testing (the reference).

**Results:**

In the healthy volunteers, pre‐ versus post‐exercise SWE showed a median velocity of 1.16 ± 0.17 versus 1.28 ± 0.22 m/s (*P* = .01). In the CECS patients, the difference was 1.11 ± 0.11 versus 1.41 ± 0.19 m/s (*P* = .06). However, there were no SWE differences between healthy volunteers and CECS patients (*P* = .14). For pre‐ and post‐exercise SHAPE, both healthy volunteers and CECS patients demonstrated significant differences (−62.9 ± 2.09 vs –58.7 ± 2.74 dB and −59.0 ± 2.96 vs −50.8 ± 2.22 dB, respectively; *P* < .002). Healthy volunteers were statistically significantly different from CECS patients (4.2 ± 2.56 vs 8.1 ± 2.12 dB; *P* = .01). The SHAPE gradient correlated with compartment pressure testing (*r* = −0.95; *P* = .01).

**Conclusions:**

These preliminary results indicate that SHAPE may become a noninvasive alternative for diagnosing CECS.

**Abbreviations**
CECS: Chronic exertional compartment syndrome
IQR: interquartile range
SWE: shear‐wave elastography
SHAPE: subharmonic‐aided pressure estimation

Chronic exertional compartment syndrome (CECS) is a condition that typically presents itself in recreational runners, elite athletes, and military recruits.^1^ The reported incidence rate of CECS in active patients presenting with exercise‐induced leg pain is 33%.^2^ CECS arises most commonly in the calf from an increased intra‐compartmental pressure, which causes impaired tissue perfusion. Curative treatment is a fasciotomy, which has a success rate of up to 95%.^2^ However, the average delay in diagnosis and subsequent treatment is 2 years; this delay has been shown to decrease the success rate of both conservative and surgical therapy.^2^, ^3^

The etiology of CECS is not well understood, but is thought to arise from volume expansion of a muscle within a noncompliant space bounded by fascia and bone, resulting in insufficient blood flow and a resultant oxygen supply and demand mismatch in that compartment.^2^, ^4^ In some cases, the physiological response may lead to a 20% increase in muscle volume.^2^ Several risk factors have been identified, including a smaller capillary density to muscle size ratio, overuse injuries, or repetitive mechanisms from microtrauma with scar formations.^4^, ^5^

The reference standard for diagnosing CECS is compartment pressure testing, which is a direct invasive measurement of intra‐compartmental pressures, with a reported sensitivity and specificity of 63% and 95%, respectively.^6^ This test is performed by inserting a handheld 18 gauge needle connected to a pressure monitor into the calf in all 4 muscle compartments (anterior, lateral, deep posterior, and superficial posterior) both pre‐ and post‐exercise. The pressure monitor measures the compartment pressure directly (in mmHg), and pre‐ to post‐exercise differences above 30 mmHg indicates CECS.^1^ Direct compartment testing is invasive, painful, and carries a small risk of bleeding and infection at the testing site. Furthermore, there is significant variability in this technique, with some studies finding more than 5 mmHg difference in 40% of compartmental pressure measurements.^7^, ^8^

Several noninvasive imaging modalities have been used to diagnose CECS. Compared to pressure testing, MRI showed similar sensitivity, but lower specificity (≤60%).^9^ While MRI has shown some diagnostic promise, it is more expensive, less ubiquitous, and less accurate than compartment testing.^8^, ^10^ Near‐infrared spectroscopy, which measures hemoglobin O_2_ saturation of tissues, has been shown to have clinically equivalent sensitivity (85%) compared to compartment testing; however, it is not readily available.^9^ Currently, an accurate, noninvasive, and cost‐effective diagnostic tool does not exist for patients with suspected CECS.

Diagnostic ultrasound, specifically shear wave elastography (SWE) and subharmonic‐aided pressure estimation (SHAPE) techniques, are safe, inexpensive, and noninvasive imaging modalities that could be used to diagnose CECS. SWE uses a higher intensity ultrasound push‐pulse to generate transversely oriented shear waves, and the velocity of these shear waves is directly proportional to tissue stiffness.^11^ Recently, the utility of SWE in musculoskeletal imaging has increased; for example, SWE is currently being used to evaluate soft tissue masses, joints, ligaments, nerves, tendons, and muscles.^11^, ^12^

The SHAPE technique utilizes microbubble‐based ultrasound contrast agents, which are safe, traverse the capillaries, and act as echo‐enhancers before dissipating from the intravascular space. The encapsulated microbubbles (<10 μm diameter) produce nonlinear oscillations at frequencies ranging from the subharmonic (ie, half of the fundamental or transmit frequency) to higher harmonics at acoustic pressures above 200 kPa.^13^ The subharmonic amplitude from (most) microbubbles has an inverse relationship with the ambient pressure, which forms the basis of SHAPE.^14^, ^15^, ^16^, ^17^, ^18^, ^19^, ^20^ Previous SHAPE clinical trials from our group and others have focused on evaluating portal hypertension, interstitial fluid pressure in breast cancer, and cardiac pressures.^14^, ^15^, ^16^, ^17^, ^20^, ^21^, ^22^ This pilot study investigated the application of SWE and SHAPE to measure intra‐compartmental muscle pressures and diagnose CECS in patients compared to healthy individuals.

## Methods

### Enrollment and Clinical Evaluation

Healthy volunteers and patients with a known diagnosis of CECS seen at the Rothman Orthopedic Institute in Philadelphia, PA (from July 2020 through January 2023) were considered for enrollment in this Institutional Review Board and Food and Drug Administration approved study (IND #112241 and NCT54105247) with all patients signing informed consent. Inclusion criteria for the patients included CECS as the primary diagnosis with no other diagnoses and age over 18. Exclusion criteria included recent trauma/surgery to the lower extremity, pregnancy, stress fractures of the lower extremity, diabetic neuropathy, peripheral vascular disease, pressure ulcers or treatment for pressure ulcers, coronary artery disease, and active pulmonary disease as well as known allergy to any components of the ultrasound contrast agent employed in this study (Definity; Lantheus Medical Imaging, N Billerica, MA).

### Exercise Protocol

All subjects (CECS patients as well as volunteers) were submitted to a standardized exercise protocol on a treadmill. This protocol consisted of running at 3.5 mph against a 3° slope for 10 min or until symptom onset (leg pain and/or fatigue). If symptoms did not occur by 10 min, running at 4 mph and/or against a 6° slope was added for an additional 5 min or until symptom onset.

### Ultrasound Examinations

The 2D grayscale ultrasound examinations were conducted within 14 days of the compartment pressure testing on either the anterior or lateral tibialis muscle (standardized as one‐third the way down the tibia) using a LOGIQ E10 ultrasound system (GE HealthCare, Waukesha, WI). The ultrasound scans were performed pre‐ and post‐exercise on the right anterior tibialis muscle in the volunteers and on the symptomatic muscle in the CECS patients with subjects lying supine on an exam table. Muscle stiffness was measured by SWE (as shear velocity in m/s) with a C1‐6 curvilinear transducer. Approximately 15–20 SWE still images were acquired for each examination and analyzed offline. For data analysis, 12 regions of interest (ROI) were placed in the middle or bottom third of an ROI (size: 2 cm) and a box size of approximately 3 × 2 cm. The median SWE velocity within the 12 ROIs was determined, and the interquartile range (IQR), IQR/median (%), and overall median SWE velocities were calculated.

**Figure 1:**
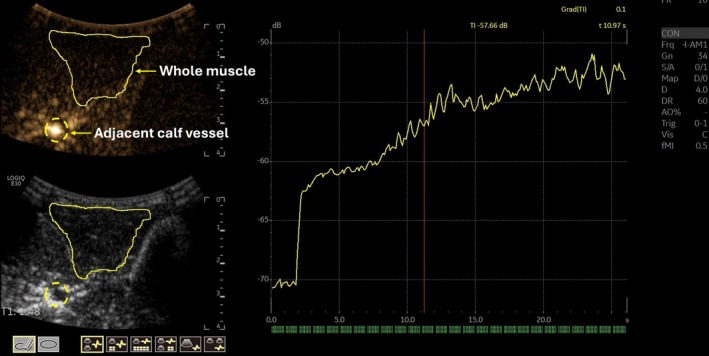
An example of SHAPE power optimization. After peak enhancement is achieved, the SHAPE optimization algorithm is initiated. The SHAPE optimization acquires subharmonic imaging from mechanical indices starting from 0.0 to 0.5 over a 26 s timeframe. This increase in acoustic output from a gradual increase from MI of 0.0 to 0.5 over 26 s generates a time intensity curve. The optimal power corresponds to the steepest slope, as this is where the subharmonic signal is most sensitive to the ultrasound contrast agents for pressure estimation. The only anatomical structure that was used to determine the power optimization was the whole muscle (solid yellow region of interest). The adjacent calf vessel (yellow dotted circle) is not included in the power optimization but is added as an example of the gradient used for SHAPE.

SHAPE imaging was conducted with a C2‐9 transducer operating in subharmonic imaging mode (transmitting at 5.8 MHz and receiving at 2.9 MHz) on a LOGIQ E10 scanner. Two vials of the ultrasound contrast agent, Definity (ie, 3 mL), were suspended in 50 mL of saline and infused intravenously at a rate of 4–10 mL/min titrated to effect through a 20 or 22 gauge angio‐catheter in a peripheral arm vein. The rate (within 4–10 mL/min) was increased until peak enhancement within the area of interest was achieved. At peak enhancement, an individual power optimization was performed pre‐exercise to determine the optimum acoustic output power for SHAPE imaging (Figure 1).^23^ Once the acoustic power was determined, the sonographer acquired 3–5 clips of 5 s duration of the subharmonic signals from the muscle. For SHAPE data analysis, an ROI was drawn around the tibialis anterior muscle for each acquired clip, and intensity values (in dB) within the ROI were exported offline and averaged. Additionally, a SHAPE gradient was calculated. This consisted of an ROI placed around the tibialis anterior muscle and adjacent calf vessel, and the difference between the two ROIs was calculated (ie, whole muscle normalized to the adjacent calf vessel). All SHAPE data analysis was performed offline.

## Statistical Analyses

Statistical analysis was performed in PRISM version 10.3.1 (GraphPad Software, Boston, MA). Paired *t*‐tests were used to compare pre‐ to post‐exercise measurements within the healthy volunteer group and the CECS group. Unpaired *t*‐tests were used to compare differences in pre‐ to post‐exercise between groups. Receiver operating characteristic curve (ROC) analysis was performed to evaluate diagnostic performance. Simple linear regression was used to compare SWE and SHAPE to the reference standard, compartment pressure testing. In this study, *P*‐values <.05 represented statistical significance.

## Results

A total of 15 subjects agreed to participate in this pilot study, including 10 healthy volunteers and 5 symptomatic patients. Imaging data were only available from 14 subjects, due to a technical failure with the ultrasound scanner in one subject from the healthy volunteer group. Hence, there were nine subjects in the healthy volunteer group and five in the CECS group. Table 1 shows patient demographics. For the healthy volunteers, the mean age was 33 ± 5.6 years, while among the CECS subjects, the mean age was 27 ± 2.4 years (*P* = .03). Five males and four females were included in the healthy control group, while the CECS group consisted of two males and three females (*P* = .47). The right calf was imaged in all 9 healthy volunteers and in 3 of the CECS participants, while the left calf was imaged in the remaining 2 CECS participants. Among the CECS subjects, four had CECS in the anterior compartment, while one subject had CECS in the lateral compartment (cf., Table 1). Regarding the exercise protocol, none of the healthy volunteers stopped exercise due to leg pain; however, there were 5 healthy volunteers that did not complete the entire exercise protocol given physical constraints, while four healthy volunteer participants completed the entire exercise protocol. None of the participants in the CECS group completed the entire exercise protocol. There were two CECS participants that had symptom onset in the first 10 min and three CECS participants that completed half the protocol until symptom onset.

**Table 1:** Patient Demographics

	Healthy Volunteers	CECS Participants
Participants	9	5
Age (mean ± SD, years)	33 ± 5.6	27 ± 2.4
Male	5	2
Female	4	3
Race		
Asian	2	0
White or Caucasian	7	2
N/A	0	2
Ethnicity		
Other Hispanic	2	0
Not Hispanic	7	2
N/A	0	2
Calf imaged		
Right	9	3
Left	0	2
Compartment imaged		
Anterior	9	4
Lateral	0	1

**Figure 2:**
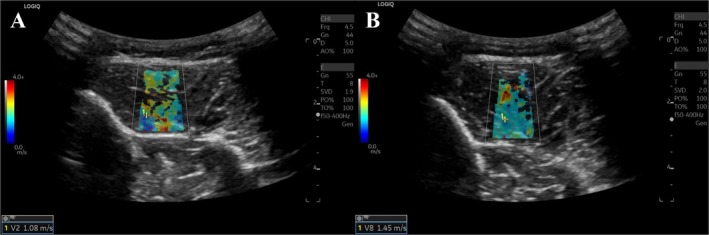
SWE measurements in a 21‐year‐old male CECS subject with left anterior calf compartment pain after exercise. The SWE imaging V Median stiffness pre‐exercise was 1.12 m/s (**A**), which increased to 1.49 m/s (**B**) immediately post‐exercise, indicating an increase in tissue stiffness.

Figure 2 is an example of a 26‐year‐old male CECS subject with bilateral pain after exercise. However, the participant had more pain in the left anterior compartment, which was selected for scanning. His symptoms have persisted for ~2 years and typically begin within 5 min of performing exercise or activity. After the exercise, the median SWE velocity increased from 1.12 m/s (Figure 1A) to 1.49 m/s (Figure 1B) indicating an increase in tissue stiffness. During the compartment pressure testing, the anterior compartment had a difference of 47 mmHg pre‐ to post‐exercise (from 15 to 62 mmHg, respectively).

In the healthy volunteers, the pre‐ versus post‐exercise SWE measurements showed a median velocity of 1.16 ± 0.19 m/s versus 1.29 ± 0.22 m/s (*P* = .01), IQR of 0.09 ± 0.04 m/s versus 0.11 ± 0.06 m/s (*P* = .06), and an IQR/median of 8.16 ± 4.1% versus 9.20 ± 3.9% (*P* = .67). In the CECS patients, the corresponding values were 1.14 ± 0.12 m/s versus 1.38 ± 0.18 m/s median velocities (*P* = .06), with an IQR of 0.06 ± 0.01 m/s versus 0.16 ± 0.08 m/s (*P* = .05), and an IQR/median of 5.66 ± 0.89% versus 10.88 ± 4.44% (*P* = .05). The difference from pre‐ to post‐exercise in median SWE velocities showed no statistically significant differences between the healthy volunteers and the CECS patients (0.13 ± 0.12 vs 0.29 ± 0.24 m/s; *P* = .14). Additionally, this SWE gradient did not correlate with the compartment pressure testing (*r* = .53; *P* = .36).

**Figure 3:**
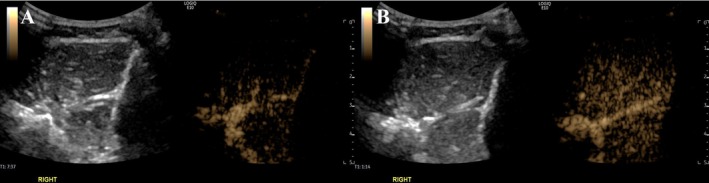
SHAPE imaging in a 28‐year‐old male CECS subject with right anterior calf compartment pain after exercise with dual grayscale ultrasound (left side of image) and subharmonic imaging (right side of image). Interestingly, in some CECS participants, there was an increase in subharmonic signal (in dB) post‐exercise, which is not fully understood at this point.

Figure 3 shows SHAPE measurements in a 28‐year‐old male CECS subject with right anterior calf compartment pain after exercise acquired in dual grayscale and subharmonic imaging modes (left and right side of the images, respectively) at an optimal mechanical index of 0.18. When comparing SHAPE results from pre‐ to post‐exercise, there was an increase in vascularity from exercise. Even though there was an increase in vascularity post‐exercise, when using the SHAPE gradient (adjacent calf vessel minus whole muscle to normalize the signal), there was an inverse relationship between SHAPE and compartment pressure testing. During the compartment pressure testing, the anterior compartment showed a difference of 50 mmHg (increasing from 25 to 75 mmHg after exercise).

**Figure 4:**
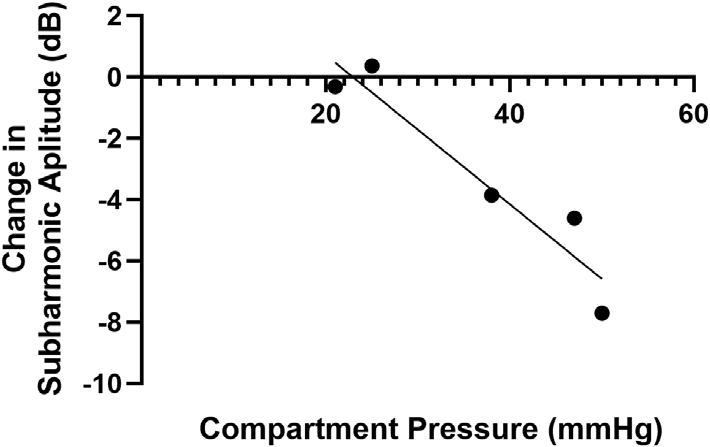
The relationship between the SHAPE gradient (the difference in whole calf muscle and the adjacent calf vessel) with the reference standard, compartment pressure testing, which shows a strong correlation of *r* = −0.95 (*P* = .01).

When evaluating the differences between the SHAPE optimal powers between healthy volunteers and CECS participants, there were no differences between groups (0.17 ± 0.05 and 0.21 ± 0.04, *P* = .30). In terms of pre to post exercise in healthy volunteers and in CECS participants, there were statistically significant differences in the muscle between pre to post exercise in the healthy volunteer group and CECS group (−62.85 ± 2.09 dB vs −58.66 ± 2.74 dB and −58.96 ± 2.96 vs −50.82 ± 2.22 dB, *P* < .002). Importantly, the SHAPE data demonstrated a statistically significant difference in the muscle between the healthy volunteers and the CECS groups with respect to the difference from pre‐ to post‐exercise (4.19 ± 2.56 vs 8.14 ± 2.12 dB, *P* = .01). ROC analysis demonstrated a Youden's index of 5.80 with an area under the ROC curve of 0.87 and 100% sensitivity and 78% specificity (95% confidence interval: 0.67–1.00, *P* = .03). This indicates that SHAPE can differentiate between normal volunteers and patients with CECS; albeit based on a small sample size. When evaluating the SHAPE gradient between healthy volunteers and patients, there was not a statistically significant difference (−0.02 ± 5.51 vs −3.23 ± 3.31 dB; *P* = .26). Encouragingly, when comparing the SHAPE gradient and the reference standard, compartment pressure testing, there was a strong correlation with an *r*‐value of −.95 (*P* = .01) as shown in Figure 4.

## Discussion

In this pilot study, we used SWE and SHAPE to assess skeletal muscle stiffness and pressure as markers for differentiating between healthy volunteers and patients with CECS. SWE and SHAPE were feasible and well tolerated in both the pre‐ and post‐exercise states for healthy volunteers and CECS subjects alike.

There were higher median SWE velocities measured pre‐ to post‐exercise in the group of healthy volunteers (*P* = .01), but not among the CECS patients (although there was a trend towards significance; *P* = .06). However, SWE velocity values could not differentiate between healthy volunteers and CECS patients in this pilot study (*P* = .14). Conversely, for the SHAPE pressure estimates, there was a statistically significant difference between normal and CECS groups from post‐ to pre‐exercise (4.19 ± 2.56 vs 8.14 ± 2.12 dB, *P* = .01), but surprisingly there was no difference when evaluating the SHAPE gradient between the two study groups (*P* = .26). Nonetheless, SHAPE measurements correlated with the compartment pressure testing (cf., Figure 4), which was expected based on prior studies using SHAPE.^14^, ^15^, ^16^, ^17^, ^19^, ^23^

SWE has been explored for use in many musculoskeletal applications, including in a first in humans study of acute compartment syndrome.^24^, ^25^ Zhang et al. reported on calf muscle stiffness in 212 healthy volunteers and 9 patients with clinical suspicion of acute compartment syndrome and were able to differentiate between surgical patients (*N* = 5) and patients undergoing more conservative treatment options (*N* = 4) based on the ratio of SWE values (in kPa) of the affected and unaffected muscle compartments (*P* < .05).^25^ However, the SWE measurements in the patients were never directly compared to those of the volunteers, and there was no reference standard verifying the compartment syndrome diagnoses.^25^ Still, the relationship between SWE and compartment testing results is generally supported by pre‐clinical studies (in a turkey model).^26^, ^27^ The study described here does have an independent reference standard (ie, compartment testing), but could not differentiate the CECS patients from the healthy volunteers using SWE. Nonetheless, taken together, these studies do seem to indicate that further investigations into the role of SWE in diagnosing CECS are warranted.

The use of CEUS in musculoskeletal applications has mainly focused on flow and perfusion parameters.^28^, ^29^, ^30^ This may be a worthwhile topic for future studies since Hargens et al^31^ showed that muscle compartment is maintained by four pressures: capillary blood pressure, capillary blood oncotic pressure, tissue‐fluid pressure, and tissue‐fluid oncotic pressure. An increase in compartment pressure caused a decrease in blood flow caused by either active compression of small arterioles or by passive collapse of capillaries when tissue pressure increases above intra‐capillary pressure. An increase in compartment pressures is expected in the majority of CECS patients who exert themselves. Although the pressure values are variable, symptomatic patients invariably have higher pressure results on compartment testing compared to healthy patients.^32^

Importantly, in this study there was an increase in perfusion post‐exercise, and when evaluating the data in absolute numbers, it does not seem to be pressure dependent. However, when evaluating the data as a gradient (eg, difference in adjacent calf vessel minus whole muscle), this relationship does appear to be pressure dependent, given the inverse relationship in subharmonic signal gradient and pressure (cf. Figure 4). These conflicting results may contribute to evaluating our study population in two different physical states (eg, before and after exercising). Traditionally, SHAPE studies have focused on one physical state. For example, participants with portal hypertension evaluating catheter‐based measurements undergoing transjugular liver biopsy or evaluating interstitial fluid pressure in breast cancer participants with breast cancer.^15^, ^16^, ^17^, ^18^ Additional research is needed to fully investigate the relationship between perfusion and SHAPE post‐exercise, and if a better reference standard is needed or the dominance in perfusion post‐exercise affects the SHAPE data.

Nonetheless, this study has several limitations that must be acknowledged. First, the study has a small sample size of healthy volunteers and in particular CECS patients. Given the uncomfortable nature of compartment testing, the CECS patients did not have their compartment pressure testing at the same time as the SWE and SHAPE examinations. Instead, imaging was performed within 14 days of the compartment pressure testing. Observations from this study require further investigation with a larger sample size in a multi‐center clinical trial and with SWE and SHAPE imaging performed nearer in time to the compartment pressure testing.

## Conclusion

The results of this pilot study indicate that SHAPE with Definity can potentially diagnose CECS in symptomatic patients; albeit based on a small sample size. A noninvasive imaging‐based alternative for diagnosing CECS may also be safer than invasive compartment pressure testing and help clinicians in patient management. A larger study is required to further validate these claims.

## Data Availability

The data that support the findings of this study are available from the corresponding author upon reasonable request.
